# Mitochondrial fission process 1 protein: a comprehensive review of its core roles in mitochondrial dynamics, disease, and therapeutic targets

**DOI:** 10.3389/fcell.2025.1646072

**Published:** 2025-09-15

**Authors:** Qingzhi Ran, Chen Gao, Chunrong Xiang, Xuanhui He, Yongkang Zhang, Yin Zhang, Hengwen Chen

**Affiliations:** 1 Guang’anmen Hospital, China Academy of Chinese Medical Sciences, Beijing, China; 2 Innovation Research Institute of Traditional Chinese Medicine, Shanghai University of Traditional Chinese Medicine, Shanghai, China

**Keywords:** mitochondrial fission process 1 protein, Mitochondria, cardiovascular disease, Tumor, drug Targets, autophagy, inflammation

## Abstract

Mitochondrial fission process 1 (MTFP1) has emerged as a central regulator of mitochondrial dynamics, playing indispensable roles in maintaining organellar integrity, bioenergetic homeostasis, and stress adaptation - particularly in high-energy-demand tissues such as cardiac and skeletal muscle. Mounting evidence implicates MTFP1 dysfunction in the pathogenesis of diverse diseases including cardiovascular disorders, myopathies, and cancer. Beyond its canonical role in mediating mitochondrial fusion-fission balance, recent studies have unveiled MTFP1’s multifaceted involvement in calcium signaling modulation, ROS metabolism, and mitochondria-ER communication networks, substantially expanding its functional repertoire in cellular physiology. The protein’s pleiotropic effects stem from its ability to integrate metabolic status with organelle dynamics and quality control mechanisms. Particularly noteworthy is MTFP1’s cell-type-specific regulation of the ROS-calcium axis, which appears critical for its differential impacts in disease states. These discoveries position MTFP1 as both a mechanistic linchpin connecting mitochondrial dynamics to cellular homeostasis and a promising but challenging therapeutic target requiring precise contextual modulation. Current research frontiers focus on elucidating tissue-specific regulatory mechanisms of MTFP1 activity, developing microenvironment-sensitive targeting strategies, and exploring its potential as a biomarker for mitochondrial dysfunction-related pathologies. This evolving understanding of MTFP1’s integrative functions opens new avenues for developing precision therapies targeting mitochondrial dynamics in energy-metabolism-linked diseases.

## Graphical Abstract

**Figure:**
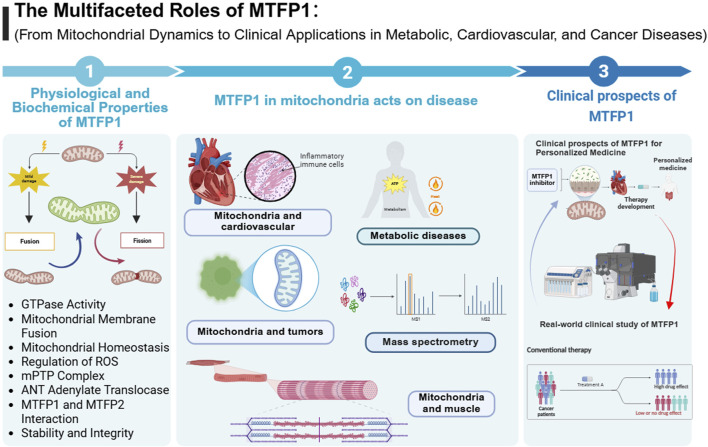
Note: ADP, Adenosine Diphosphate; ATP, Adenosine Triphosphate; MTFP1, mitochondrial fission process 1.

## 1 Introduction

The mitochondrial fission process 1 (MTFP1) protein plays a pivotal role in regulating cellular energy metabolism and maintaining mitochondrial dynamics (Hu et al., 2021). Mitochondrial dynamics represents fundamental mechanism by which cells adapt to a variety of stressful conditions, particularly when faced with energy deficits caused by reduced oxygen or glucose concentrations (Ruiz et al., 2021). In the event of mitochondrial dysfunction or exposure to metabolic toxicants such as reactive oxygen species (ROS) and the cytochrome C complex (Cyt C), MTFP1 facilitates mitochondrial fusion to maintain structural integrity, thereby ensuring cell viability and function (Aranda-Rivera et al., 2021; Tong et al., 2020). In mammalian cells, MTFP1 expression and function are controlled by several highly conserved signaling pathways that are rapidly activated in response to a decline in intracellular ATP levels (Naviaux, 2014). AMP-activated protein kinase (AMPK), which serves as the primary sensor of cellular energy status, undergoes complete phosphorylation and activation when oxidative phosphorylation (OXPHOS) is impaired. This modulates a number of processes, including lipid metabolism, glucose metabolism, mitochondrial autophagy, and the mammalian target of rapamycin complex 1 (mTORC1) signaling pathway (Valero, 2014). It is proposed that MTFP1 occupies a pivotal role in these energy regulatory pathways and interacts with the endoplasmic reticulum to modulate calcium homeostasis, participate in stress responses, and determine cell fate (Jin et al., 2022; Rabinovich-Nikitin et al., 2021). Prolonged nutrient deficiency and energy stress result in transcriptional alterations in a multitude of metabolic processes, with MTFP1 functioning as a guardian of mitochondrial health, thereby ensuring cell viability under adverse conditions (Liu et al., 2024). Dysfunctional MTFP1 not only leads to mitochondrial fragmentation but can also trigger oxidative stress and cell death through the overproduction of ROS and dysregulation of calcium (Song et al., 2021). It is noteworthy that the regulatory functions of MTFP1 are not limited to a single cell type or pathological condition. Its role in cardiovascular disease, cancer and skeletal muscle diseases is diverse and exhibits bidirectional complexity (Hombrebueno et al., 2019).

**FIGURE 1:**
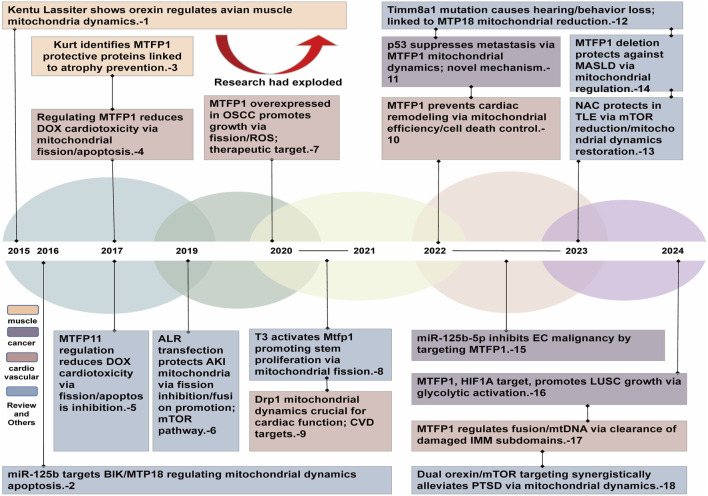
MTFP1 studies the historical course chart from 2015 to 2024. MTFP1, Mitochondrial fission process 1; DoX, Doxorubicin; BiK, Bcl-2-like protein 11; ALR, Adenosine L-Ribose; AKI, Acute Kidney Injury; HK, Hexokinase; mTOR, mammalian target of rapamycin; BP1, Mitochondria-Related Biomarker 1; ROS, Reactive Oxygen Species; OSCC, Oral Squamous Cell Carcinoma; PrP1, Prion Protein 1; EPK1, Extracellular Signal-Regulated Kinase 1; PTSD, Post-Traumatic Stress Disorder; NAC, N-Acetylcysteine; EC, Endothelial Cell.

Recent studies have increasingly elucidated the diverse roles of MTFP1 in various pathological contexts. For example, within the cardiovascular system, MTFP1 has been demonstrated to protect myocardial cells from ischemia-reperfusion injury by modulating mitochondrial fusion (Tian et al., 2022; Wang et al., 2020). In the context of cancer, MTFP1 expression may be closely linked to tumor invasiveness and drug resistance, thereby offering new insights into the potential of cancer therapies targeting MTFP1 (Choi and Han, 2021). Furthermore, MTFP1 exerts a protective role in neurodegenerative diseases by regulating mitochondrial autophagy, which reduces the accumulation of toxic proteins in neurons and delays disease progression (Panchal and Tiwari, 2019). Given the centrality of MTFP1 in cellular energy metabolism and stress responses, as well as its central role in a number of diseases, this review presents a systematic catalog of the research trajectory and key milestones of MTFP1 for the first time (Figure 1) (Teodorof-Diedrich and Spector, 2018). The objective is to provide a comprehensive summary of the physiological mechanisms involved in mitochondrial biology and the pathological mechanisms underlying various diseases, thus establishing a foundation for the future development of MTFP1-targeted pharmacotherapies. The ultimate objective is to enhance the prognosis for patients afflicted with diseases associated with mitochondrial dysfunction (Cen et al., 2021).

## 2 Physiological and biochemical properties of MTFP1

MTFP1 is a central protein located on the inner mitochondrial membrane and is widely expressed in various cell types, including cardiomyocytes, skeletal muscle cells, and neurons (Chen and Chan, 2009; Rangaraju et al., 2019; Roca-Portoles and Tait, 2021). MTFP1, a GTPase localized to the inner mitochondrial membrane (IMM) (Celardo et al., 2014). Mitochondrial fusion and fission are fundamental processes that maintain the homeostasis of mitochondrial morphology and function, which are critical for cell survival and energy metabolism (Sas et al., 2007; Valero, 2014). MTFP1 facilitates the interaction of mitochondrial outer membrane proteins through GTP hydrolysis, thereby promoting mitochondrial outer membrane fusion and the formation of a larger, more functional mitochondrial network (Bastian et al., 2019). An understanding of the structure and function of MTFP1 is essential for elucidating its role in cellular physiological and pathological processes (Wang et al., 2023b).

### 2.1 Structural properties of MTFP1

MTFP1, a major protein of the inner mitochondrial membrane, is primarily structured to include transmembrane domains that embed it into the inner mitochondrial membrane (Iorio et al., 2021). It interacts with other components of the mitochondrial permeability transition pore (mPTP) complex, thereby regulating membrane permeability and mitochondrial respiratory function (Park et al., 2018). From a structural perspective, MTFP1 forms a complex with other mitochondrial proteins that is indispensable for maintaining the integrity of the mitochondrial inner membrane (White et al., 2017). MTFP1 has multiple transmembrane domains that facilitate its stable anchoring within the inner mitochondrial membrane. Specifically, the transmembrane domains of MTFP1 consist of a series of alpha helices that span the double-layered phospholipid structure of the inner membrane, thereby anchoring different parts of the protein on either side of the membrane (Kulkarni et al., 2023; Ragunathan et al., 2022). Through these transmembrane domains, MTFP1 interacts with the lipid environment of the inner membrane, thereby maintaining its integrity and regulating mitochondrial permeability and respiratory function (Morris et al., 2024). The MTFP1 protein is composed of multiple domains, including an N-terminal GTP-binding domain, transmembrane domains, and a C-terminal dimerization domain (Sun et al., 2024). The GTP-binding domain, located at the N-terminus of MTFP1, is accountable for GTP binding and hydrolysis and serves as the driving force behind mitochondrial outer membrane fusion (Colombini, 2017). The transmembrane domains serve to anchor MTFP1 to the outer membrane, thereby facilitating its effective participation in the membrane fusion process (West et al., 2016). The C-terminal dimerization domain enables the MTFP1 protein to form homodimers or heterodimers, with the latter pairing with MTFP2 proteins. This dimerization is essential for the effective fusion of the mitochondrial outer membrane (Ji and Kang, 2015; Luchetti et al., 2022).

MTFP1 protein is widely expressed across various tissues, including the heart, liver, brain, skeletal muscle, and colon (Rey et al., 2020; Sim et al., 2012). Gene expression analysis (Table 1) reveals significant differences in MTFP1 RNA levels across different tissues. In Table 1, MTFP1 expression is presented in human tissue structures. For instance, MTFP1 levels are relatively higher in metabolically active tissues, such as the liver and skeletal muscle; however, elevated expression is also observed in the colon, a tissue not typically regarded as high-metabolism. This variability may reflect the diverse demands for mitochondrial dynamics in different tissues, which not only involve energy metabolism but also cell turnover, barrier functions, and signaling regulation (Boscolo et al., 2013). The MTFP1 gene is situated within the 3p25.2 region of the human chromosome and encompasses 17 exons. The protein encoded by this gene is involved in the fusion process of the mitochondrial outer membrane and is responsible for maintaining the function and morphology of the mitochondria (Sabat et al., 2021). The protein structure of MTFP1 comprises several functional domains, including a GTPase domain that is responsible for GTP hydrolysis, transmembrane domains, and a C-terminal domain that is responsible for protein dimerization (Szabadkai et al., 2004). These domains collectively facilitate the fulfillment of MTFP1’s distinctive biological functions within the cell. The GTPase domain of MTFP1 is situated at the N-terminus (amino acid positions 101–300) and functions to induce conformational alterations in mitochondrial outer membrane proteins through the binding and hydrolysis of GTP, thereby facilitating membrane fusion. The transmembrane domains are situated in the central region of the protein (amino acid positions 400–500) and serve to anchor MTFP1 to the mitochondrial outer membrane, thereby enabling its effective participation in mitochondrial fusion. The C-terminal dimerization domain (amino acid positions 600–750) enables MTFP1 to form dimers or oligomers with itself or its homologous protein MTFP2, which is essential for the effective fusion of mitochondrial membranes (Golla et al., 2024). Furthermore, the MTFP1 protein contains multiple domains with unknown functions that may potentially regulate other cellular signaling pathways, such as apoptosis and autophagy.

**TABLE 1:** RNA sequence analysis of MTFP1 in 27 human tissues.

Sample	BioSample	RPKM	Count
Adrenal	3 samples	3.121 ± 0.358	44,693
Appendix	3 samples	4.722 ± 0.144	60,531
Bone marrow	4 samples	3.691 ± 1.712	978,646
Brain	3 samples	1.4 ± 0.521	24,268
Colon	5 samples	5.185 ± 1.44	190,645
Duodenum	2 samples	6.383 ± 0.117	56,011
Endometrium	3 samples	2.386 ± 1.209	41,102
Esophagus	3 samples	2.912 ± 0.696	68,073
Fat	3 samples	0.794 ± 0.252	11,420
Gall bladder	3 samples	3.59 ± 1.274	85,155
Heart	4 samples	3.476 ± 1.771	112,020
Kidney	4 samples	5.231 ± 0.462	89,070
Liver	3 samples	2.844 ± 0.503	51,457
Lung	5 samples	1.958 ± 0.583	48,101
Lymph node	5 samples	5.669 ± 1.061	233,527
Ovary	2 samples	0.741 ± 0.011	13,876
Pancreas	2 samples	0.677 ± 0.194	12,909
Placenta	4 samples	5.953 ± 4.365	203,435
Prostate	4 samples	4.877 ± 0.931	93,197
Salivary gland	3 samples	1.016 ± 0.146	30,405
Skin	3 samples	0.957 ± 0.13	24,568
Small intestine	4 samples	4.052 ± 0.662	84,368
Spleen	4 samples	3.92 ± 0.694	105,304
Stomach	3 samples	5.081 ± 2.251	107,541
Testis	7 samples	1.513 ± 0.402	93,234
Thyroid	4 samples	3.442 ± 1.096	125,481
Urinary bladder	2 samples	4.209 ± 0.284	65,111

RPKM, reads per kilobase of exon model per million mapped reads.

### 2.2 Function of MTFP1 in mitochondrial fusion

MTFP1’s GTPase activity is a core functional element in mediating mitochondrial fusion. Mitochondrial fusion is a sequential process that requires the coordination of both the inner and outer membranes. Outer membrane fusion is primarily mediated by mitochondrial fusion proteins 1 and 2 (MFN1/2), which anchor adjacent mitochondria and promote outer membrane mixing (Chen et al., 2024; Wu et al., 2024). Inner membrane fusion, on the other hand, is driven by the dynamin-related GTPase OPA1, which undergoes GTP-dependent conformational changes to remodel and fuse the inner membrane (Szabadkai et al., 2004). Although MTFP1 is anchored to the outer mitochondrial membrane, recent evidence suggests that it can indirectly influence inner membrane fusion by modulating mitochondrial energy metabolism and membrane lipid composition (Rey et al., 2020; Xavier and Martinou, 2021). By maintaining mitochondrial membrane potential and stabilizing the lipid microenvironment, MTFP1 preserves the structural integrity of OPA1 and facilitates its proteolytic processing into long and short isoforms—critical for efficient inner membrane fusion (Li et al., 2025). This functional link positions MTFP1 as a potential coordinator between outer membrane fusion events (MFN1/2) and inner membrane remodeling (OPA1), thereby ensuring mitochondrial network continuity and bioenergetic homeostasis. In addition to its role in membrane fusion, MTFP1 also regulates mitochondrial function through the mitochondrial permeability transition pore (mPTP) complex. Its interactions with adenine nucleotide translocator (ANT) and cyclophilin D (CypD) control inner membrane permeability, maintain membrane potential, and prevent abnormal pore opening, thus avoiding mitochondrial depolarization and cell apoptosis. These dual functions in fusion regulation and permeability modulation make MTFP1 a multifunctional regulatory hub for mitochondrial homeostasis (Montes de Oca Balderas and Aguilera, 2015).

**FIGURE 2:**
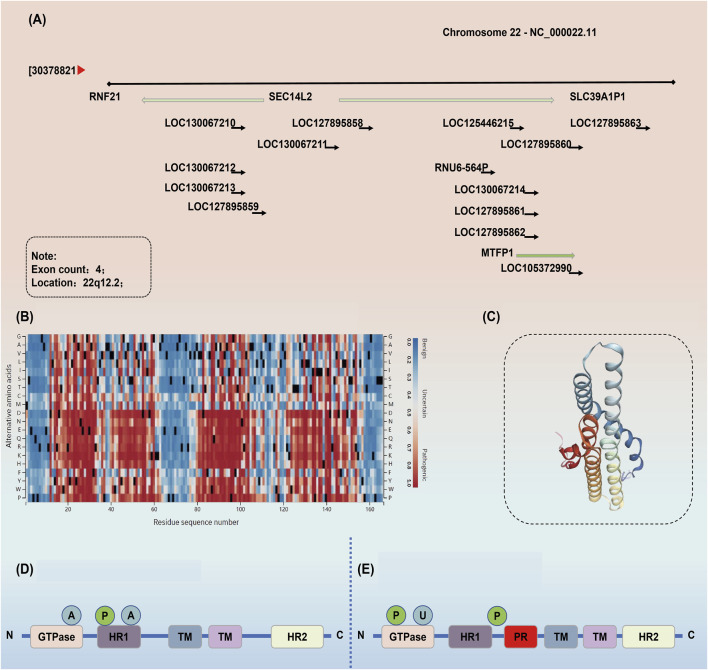
Physiology and biochemistry of MTFP1. **(A)** Genomic context of MTFP1. **(B)** AlphaMissense Pathogenicity Heatmap of MTFP1. **(C)** Biological Assembly of MTFP1. **(D)** Fusion Factor of MTFP1. **(E)** Fusion Factor of MTFP2. Abbreviations: HR1, Hemagglutinin Receptor 1; TM, Transmembrane.

**FIGURE 3:**
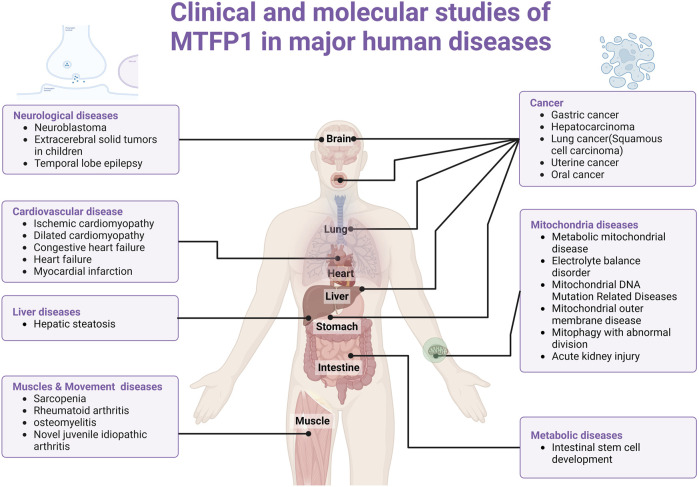
The clinicopathological diagram of MTFP1 in various tissues and organs of the human body. Abbreviations: MTFP1, Mitochondrial fission process 1; Drp1, Dynamin-related protein 1; FIS1, Fission 1 Protein; OMM, Outer Mitochondrial Membrane; IMM, Inner Mitochondrial Membrane; SMEM, Small Molecule Exchange Membrane; HSCTe, Human Stem Cell Transplantation; CMA, Chaperone-Mediated Autophagy.

The GTPase activity of MTFP1 is acritical component of its function. By binding and hydrolyzing guanosine triphosphate (GTP), MTFP1 drives the conformational change of the inner mitochondrial membrane proteins, which ultimately results in the binding and subsequent fusion of the two inner mitochondrial membranes (Pang et al., 2024). This process is vital for the maintenance of mitochondrial homeostasis, particularly in energy-demanding human tissues such as cardiac and skeletal muscle (Wisowski et al., 2022). The regulatory role of the MTFP1 protein is not limited to the process of physical fusion; it also encompasses the regulation of mitochondrial function (modulating mitochondrial inner membrane function, respiratory chain activity, and the antioxidant system). This includes the regulation of the spatial distribution of mitochondrial DNA, the maintenance of mitochondrial membrane potential, and the regulation of ROS (Kruglik et al., 2017). MTFP1 regulates mitochondrial bilayer membrane permeability and mitochondrial energy metabolism through interaction with other components of the mPTP complex (Prastya et al., 2020). Specifically, MTFP1 binds to the mPTP complex and influences the opening and closing of mPTP, thereby regulating homeostasis in both directions (Matija et al., 2023). Upon activation, MTFP1 facilitates the maintenance of the mPTP shutdown state, thus preserving the electrochemical gradient of the mitochondrial membrane, promoting normal respiratory function, and ensuring the homeostasis of mitochondrial energy metabolism (Montagnani Marelli et al., 2024). This process serves to prevent uncoupling and increased permeability of the mitochondrial membrane, thereby preventing apoptosis from occurring. MTFP1 interacts with several key components of the mPTP complex, including adenylate translocase (ALT), cyclic adenosine monophosphate-dependent phosphokinase (CypD), and others (Scarpelli et al., 2019; Volkov and van Nuland, 2012). These interactions regulate the state of mPTP by forming a stable complex that controls the permeability of mitochondrial membranes (Shneyer et al., 2017). Upon binding to proteins such as ANT and CypD, MTFP1 inhibits the aberrant opening of mPTP, thereby preventing mitochondrial depolarization and reduced ATP synthesis (Zhou et al., 2017). The formation of this complex is of great importance for the maintenance of the integrity of the inner mitochondrial membrane and prevents the occurrence of apoptosis (Montes de Oca Balderas and Aguilera, 2015). MTFP1 and MTFP2 are homologous proteins that are involved in the fusion process of the inner mitochondrial membrane (Xavier and Martinou, 2021). Despite their analogous domains and functions, their roles in particular cells and tissues may diverge. For example, MTFP2 is involved in not only in mitochondrial fusion but also in the formation of endoplasmic reticulum contact sites, which regulate the transmembrane transport of calcium ions and lipids (Hensen et al., 2019; Singh et al., 2018). Consequently, the cooperative interaction between MTFP1 and MTFP2 represents a pivotal mechanism for maintaining intracellular mitochondrial homeostasis. Elucidating the cooperative mechanism of the two will greatly contribute to the understanding of the intricate regulation of mitochondrial dynamics (Figures 2, 3).

## 3 The role of MTFP1 in mitochondria: from mitochondrial fission to mitophagy

As a crucial GTPase in the inner mitochondrial membrane, MTFP1 is essential for maintaining mitochondrial morphology and function. Mitochondrial fusion and fission play a pivotal role in cellular energy metabolism and exert a profound influence on mitophagy, cell fate determination, and intracellular signaling (Chen et al., 2023). MTFP1 plays a pivotal role in cellular adaptation to diverse stress conditions by modulating the structure and function of the mitochondrial network (Rovira-Llopis et al., 2017). As a central regulator of mitochondrial energy metabolism, MTFP1 plays a pivotal role in the formation and functionalization of mitochondrial functional networks, thereby promoting mitochondrial fusion, which is essential for ATP production and maintenance of cellular homeostasis (Wang et al., 2023a). Mitochondria serves as the primary site of cellular energy production, with their oxidative phosphorylation pathway accounting for the majority of ATP synthesis within the cell (Shi et al., 2021). Mitochondrial energy metabolism homeostasis is of particular importance in tissues with high energy requirements, such as the heart and skeletal muscle (Zhong et al., 2023). c (Zhao et al., 2021). An increase in ROS levels not only triggers cellular oxidative stress but also causes mitochondrial DNA damage, impairing mitochondrial physiological and metabolic functions. Elevated ROS production disrupts mitochondrial homeostasis, damaging energy production and contributing to the development of various pathologies. Importantly, the extent of ROS accumulation and its detrimental effects may vary depending on the physiological context, such as during acute physiological stress or in chronic disease states (Chen et al., 2023). This underscores the need for a comprehensive understanding of ROS’s role in cellular homeostasis under different conditions (Yang et al., 2023). Consequently, MTFP1 is of vital importance in maintaining mitochondrial health and the stability of cellular energy metabolism.

### 3.1 Coordination mechanism of mitochondrial fission and fusion

**FIGURE 4:**
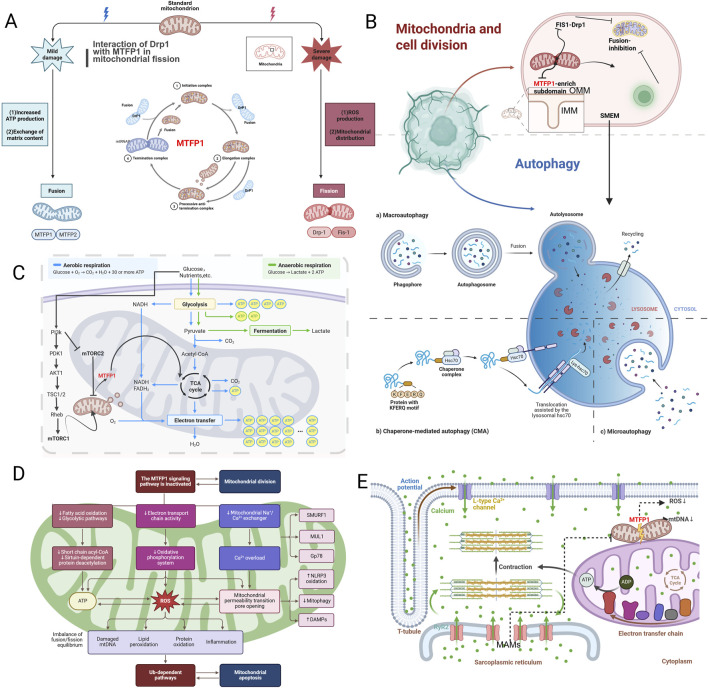
MTFP1 and mitochondria. **(A)** MTFP1 and Mitochondrial Fission-Fusion Balance; **(B)** Mitochondria and cell division; **(C)** MTFP1 and Mitochondrial Energy Metabolism; **(D)** MTFP1 and ROS; **(E)** MTFP1 and Intracellular Electron Transport Chain. Abbreviations: PI3k, Phosphoinositide 3-Kinase; PDK1, 3-Phosphoinositide-Dependent Protein Kinase 1; AKT, RAC Alpha Serine/Threonine-Protein Kinase; TSC1, Tuberous Sclerosis Complex 1; RhEb, Ras Homolog Enriched in Brain; mTORC, Mammalian Target of Rapamycin Complex; NADH, Nicotinamide Adenine Dinucleotide, Reduced Form; FADH2, Flavin Adenine Dinucleotide; ATP, Adenosine Triphosphate.

Mitochondrial fusion and fission are highly coordinated processes that are co-regulated by proteins such as MTFP1 and dynamin-related protein 1 (Drp1) (Yoo and Jung, 2018). MTFP1 is primarily responsible for the fusion of the inner mitochondrial membrane, whereas Drp1 mediates the regulation of mitochondrial fission (Roca-Portoles and Tait, 2021). Mitochondrial fission serves not only to distribute mitochondria to isolated daughter cells during cell division, but also plays a pivotal role in the removal of damaged mitochondria and the regulation of mitochondrial number (Reddy and Oliver, 2019). MTFP1 plays a pivotal role in maintaining mitochondrial network homeostasis. It does so by promoting mitochondrial fusion and preventing excessive mitochondrial fragmentation, which in turn alleviates the inflammatory response in the intracellular environment (Wu et al., 2022a). This equilibrium enables cells to respond to external stimuli and adapt to diverse physiological requirements, as illustrated in Figure 4A. The mitochondrial membrane is comprised of two distinct layers: the outer membrane (OMM) and the inner mitochondrial membranes (IMMs). The IMM projects into the mitochondrial genome, which contains protein-DNA complexes in the matrix. mtDNA encodes for 13 core subunits of the respiratory chain, which are essential for maintaining the basic metabolic processes of cells (Yu et al., 2020). The maintenance of cellular homeostasis requires the presence of only two copies of nuclear DNA (nDNA), whereas the number of mitochondrial DNA (mtDNA) copies is much higher and varies considerably between cell types, typically ranging from several hundred to several thousand per cell, with higher copy numbers observed in energy-demanding tissues such as cardiac and skeletal muscle (Xie et al., 2022). MTFP1 is an integrated IMM egg self that acts on dynein-associated egg-derived cleavage factors upstream of Drp1 and is associated with the activation of mTORC1, cell death, cancer progression, and mitochondrial bioenergetics. The research team led by Prof. Wang investigated the regulation of mitochondrial morphology by MTFP1 and discovered the mechanism by which MTFP1 coordinates the inhibition of IMM fusion and OMM fission to promote peripheral mitochondrial division (Wang et al., 2017b). The loss of fusion activity of MTFP1 ensures the separation and subsequent autophagic degradation of the IMM substructure, as illustrated in Figure 4B.

#### 3.1.1 Mitochondrial fusion and fission-related factors

Mitochondrial fusion occurs in two distinct steps: outer mitochondrial membrane fusion (OMM fusion) and inner mitochondrial membrane fusion (IMM fusion). OMM fusion is mediated by Mitofusin 1 (MFN1) and Mitofusin 2 (MFN2), which interact through their transmembrane regions with the outer membranes of adjacent mitochondria, forming a complex. Additionally, MFN2 plays a role in the physical interaction between mitochondria and the endoplasmic reticulum (ER), as well as in calcium signaling exchange (Guo et al., 2023). IMM fusion is mediated by Optic Atrophy Protein 1 (OPA1), located in the IMM. OPA1 functions through the coordinated action of its long (L-OPA1) and short (S-OPA1) isoforms, promoting inner membrane fusion and maintaining the stability of mitochondrial cristae, thereby supporting efficient oxidative phosphorylation (OXPHOS) (Silva Ramos et al., 2019). Existing evidence suggests that MTFP1 deletion leads to severe mitochondrial fragmentation, accompanied by disruption of the OPA1-related fusion process (Klemmensen et al., 2024). This phenotypic association suggests that MTFP1 may indirectly influence OPA1 processing and function by regulating membrane potential (ΔΨm) or lipid environment, although direct protein interaction evidence remains lacking.

Mitochondrial fission primarily relies on the recruitment and assembly of Drp1 in the cytoplasm. The localization of Drp1 is dependent on several anchoring proteins on the outer membrane, including Mitochondrial Fission 1 Protein (FIS1), Mitochondrial Fission Factor (MFF), and Mitochondrial Dynamics Proteins of 49 kDa (MID49) and MID51. These proteins recruit Drp1 to the fission sites via specific Drp1-binding domains and coordinate localization with the contact points of the endoplasmic reticulum (mitochondria-associated membranes, MAMs) (Kerr et al., 2017). Additionally, Drp1 oligomerization to form ring-like structures is a crucial step in fission, and the final scission may also require the involvement of GTPases such as Dynamin 2 (DNM2) (Wei et al., 2017). MTFP1 was initially identified as a key factor promoting mitochondrial fission, and its absence results in reduced Drp1 assembly efficiency on the outer membrane, thereby inhibiting the fission process (Iorio et al., 2021). However, similar to its relationship with OPA1, there is currently no direct evidence of stable physical interaction between MTFP1 and Drp1 or its anchoring proteins (FIS1, MFF, MID49/51). A possible hypothesis is that MTFP1 indirectly facilitates efficient Drp1 assembly by modulating the membrane characteristics at the local fission sites.

### 3.2 Functional regulation of MTFP1 in mitophagy

Mitochondrial adaptation to metabolic states is achieved through a coordinated process of fission and fusion. Mitochondrial dynamics, including these processes, are essential for maintaining mtDNA integrity and quality control. Damaged organelles undergo fragmentation, generating smaller mitochondria that are primed for autophagic degradation (Silva Ramos et al., 2019). The severity of the mitochondrial injury influences whether these fragmented mitochondria are integrated into a healthy mitochondrial network or are eliminated through autophagy (Oshima et al., 2021). Mitophagy, the selective degradation of damaged or dysfunctional mitochondria, is crucial for maintaining mitochondrial quality (Guo et al., 2023).

MTFP1 plays a dual role in regulating both mitochondrial fusion and fission. Under normal conditions, MTFP1 promotes mitochondrial fusion, delaying the need for autophagy by facilitating the integration of partially damaged mitochondria into the existing mitochondrial network (Miller and Thorburn, 2021). However, when mitochondrial damage becomes irreversible, specific signaling pathways inhibit MTFP1 function, promoting mitochondrial fission. For example,: PINK1/Parkin pathway, Drp1 stress signaling pathway, ROS oxidative stress signaling pathway and so on. This increased fragmentation allows damaged mitochondria to be sequestered and directed to the autophagic pathway for degradation (Klemmensen et al., 2024). The dynamic regulation of MTFP1 ensures efficient mitochondrial quality control and is essential for cellular viability during stress (see Figures 4B–D).

MTFP1’s regulatory role extends beyond mitochondrial dynamics, influencing the autophagic process and inter-organelle interactions. The activity of MTFP1 is modulated by intracellular signaling molecules, including calcium levels, energy status, and post-translational modifications (Han et al., 2023b). Notably, AMP-activated protein kinase (AMPK), as a cellular energy sensor, phosphorylates MTFP1 under energy deficiency, modulating its role in both mitochondrial fusion and mitophagy (Kerr et al., 2017). Additionally, MTFP1 has been implicated in regulating calcium ion transport and lipid metabolism by participating in the formation of mitochondrial-associated membranes (MAMs), which are critical sites of inter-organelle communication between the mitochondria and endoplasmic reticulum (Kimura et al., 2017). MAMs play an essential role in maintaining cellular homeostasis and regulating signaling pathways (Dhapola et al., 2021). Calcium signaling within MAMs is intricately linked to mitochondrial function, with MTFP1 modulating mitochondrial behavior and influencing cell fate decisions by regulating these junctions (Yan et al., 2020).

In cardiomyocytes, apoptosis and autophagy are critical for maintaining cardiac function, and mitochondria play a central role in both processes. MTFP1 modulates the selectivity and efficiency of mitophagy by promoting mitochondrial fusion, thereby ensuring effective clearance of damaged mitochondria (Wei et al., 2017). When mitochondrial damage becomes irreversible, the absence of MTFP1 leads to excessive mitochondrial fragmentation, impairing mitophagy and promoting the accumulation of dysfunctional mitochondria, which increases the risk of apoptosis (Lizama and Chu, 2021). Thus, regulating MTFP1 activity can enhance mitophagy, reduce the accumulation of damaged mitochondria, and protect cardiomyocytes from apoptosis, improving overall cardiac function (see Figure 4E). MTFP1 interacts with specific proteins involved in mitochondrial fission and fusion, such as Drp1, which is essential for mitochondrial division. By binding to Drp1, MTFP1 helps regulate the balance between mitochondrial fusion and fission, influencing the overall mitochondrial network structure. Additionally, MTFP1 interacts with the outer mitochondrial membrane protein OPA1, which regulates mitochondrial fusion, further supporting its role in the maintenance of mitochondrial integrity and function. These interactions are crucial for ensuring that mitochondrial dynamics are appropriately regulated in response to metabolic demands and cellular stress.

### 3.3 MTFP1 acts on immune cells and immunomodulates through mitochondria

The function of immune cells is contingent upon the energy supply of mitochondria and the production of ROS, which are closely related to the morphological and functional status of mitochondria (Rabinovich-Nikitin et al., 2021). MTFP1 plays a pivotal role in preserving the integrity of the mitochondrial network by regulating mitochondrial fusion. This ensures that immune cells are able to provide sufficient energy and produce the appropriate amount of ROS in response to pathogens or other immune stimuli (Zhou et al., 2022). Studies have demonstrated that the dysfunction of MTFP1 in immune cells, such as macrophages and T cells, results in the fragmentation of mitochondria. This fragmentation can impair the energy metabolism capacity of these cells, potentially leading to excessive generation of reactive oxygen species (ROS). Under acute stress conditions, excess ROS may trigger abnormal inflammatory responses; in contrast, under chronic or prolonged exposure conditions, the sustained accumulation of ROS is associated with exacerbated inflammation and the promotion of progressive tissue damage (Lizama and Chu, 2021). The immune system’s normal function depends on coordination between immune cells, and MTFP1 plays an important regulatory role in this process. In the initial stage of the immune response, MTFP1 ensures that immune cells can be effectively activated and proliferated by regulating the morphology and function of mitochondria (Palikaras et al., 2015). During T cell activation, MTFP1-mediated mitochondrial fusion play a crucial role in maintaining intracellular calcium homeostasis and supporting ATP production, which in turn directly impacts T cell proliferation and effector function (Liu et al., 2021a). Furthermore, MTFP1 influences the polarization state of macrophages by regulating ROS production and inflammatory signaling pathways, thereby determining whether they tend to promote the inflammatory cytokine M1 or the anti-inflammatory factor M2 (Ma et al., 2023).

### 3.4 Association between MTFP1 and inflammatory response and chronic inflammatory diseases

The inflammatory response, defined as the body’s protective response to infection, injury, or other stressful conditions, can be considered a vital physiological process. However, excessive or persistent inflammation can lead to the development of a chronic disease (Singh et al., 2021). MTFP1 plays a dual regulatory role in the inflammatory response. First, MTFP1 reduces the overproduction of ROS by maintaining mitochondrial fusion, thereby limiting the intensity of the inflammatory response (Grimm et al., 2014). Secondly, MTFP1 influences calcium ion signaling by regulating the formation of mitochondria-endoplasmic reticulum contact sites (MAMs), which has an important influence on immune cell activity during inflammatory responses (Aggarwal et al., 2024). Studies have found that loss of MTFP1 function leads to a decrease in endoplasmic reticulum-mitochondria contact and disruption of calcium ion signaling, which in turn promotes the release of inflammatory factors and worsens the inflammatory response (Podor et al., 2015). The dysfunction of MTFP1 plays a role in the pathogenesis of various chronic inflammatory diseases (Duroux-Richard et al., 2016). For example, in rheumatoid arthritis and inflammatory bowel disease, reduced MTFP1 expression or function leads to impaired mitochondrial oxidative stress of immune cells, further exacerbating the runaway inflammatory response (Wei et al., 2018). Therefore, restoring or improving the function of MTFP1has the potential to alleviate the inflammatory response in these diseases and improve patient outcomes (Tábara et al., 2014). Modulating the function of MTFP1 by small molecule drugs or gene therapies can effectively restore mitochondrial kinetic balance, thereby inhibiting excessive inflammatory responses and improving immune function (Zhao et al., 2018). The targeting of MTFP1 to reduce ROS production and the release of inflammatory factors in macrophages is a promising new approach for the treatment of chronic inflammatory diseases (Soranno et al., 2016). Moreover, the restoration of the function of mitochondrial and endoplasmic reticulum contact sites, which are regulated by MTFP1, has the potential to establish a novel therapeutic approach for the regulation of immune cell activity and the amelioration of inflammatory responses.

## 4 The role of MTFP1 in the cardiovascular system: from mitochondrial function to cell fate

The role of MTFP1 in the cardiovascular system is a subject that merits close examination. Its function extends beyond the mere regulation of mitochondrial fusion to encompass the regulation of energy metabolism, calcium homeostasis, stress response, and pivotal biological processes such as apoptosis and autophagy in cardiomyocytes (Liu et al., 2020). Its dysfunction has been linked to the development of various cardiovascular diseases, suggesting that MTFP1 may serve as a therapeutic target for the treatment of these diseases. A comprehensive understanding of the mechanisms by which MTFP1 functions in the cardiovascular system promises not only to advance fundamental research but also to provide a valuable reference point for clinical interventions (Wang et al., 2022).

### 4.1 The central role of MTFP1 in cardiomyocyte energy metabolism

The elevated metabolic demands of cardiomyocytes underscore the significance of mitochondrial function (includes oxidative phosphorylation-driven ATP production processes and also covers the ability to maintain energy metabolism homeostasis through network structure). MTFP1 has been shown to optimize the ATP production process by promoting mitochondrial fusion and ensuring the integrity of the mitochondrial network (Ahmad et al., 2019). MTFP1-mediated membrane fusion within the mitochondria plays a pivotal role in maintaining the homogeneity of mitochondrial DNA and ensuring the efficient operation of the oxidative phosphorylation process (Dorn, 2010). This is of particular importance for cardiomyocytes to maintain their energy supply underconditions of high stress. Studies have demonstrated that the absence or dysfunction of MTFP1 results in mitochondrial fragmentation, which is associated with disorders of energy metabolism, leading to deterioration of cardiomyocyte function and death. This process plays a pivotal role in the pathological development of heart failure, as illustrated in Figure 4C (Wu et al., 2017). Professor Erminia Donnarumma’s research team has determined that MTFP1 is critical for cardiac structure and function, and their prior study revealed that constitutive silencing of MTFP1 in mouse cardiomyocytes results in fatal adult-onset dilated cardiomyopathy with expanded mitochondrial and cardiac remodeling during the transition to the heart leads to failure (Marin-Garcia and Akhmedov, 2016). Prior to the onset of the disease, the knockout of cardiac mitochondria exhibited specific defects in the IMM, futile proton egress dependent on adenine nucleotide translocase, and increased sensitivity to mitochondrial permeability transition pore opening. These findings indicate that MTFP1 undergoes physical and genetic interactions with mitochondrial permeability transition pores, which has led to the identification of new functions of MTFP1 in controlling bioenergetic efficiency and susceptibility to cell death, and has underscored its importance in preventing pathogenic cardiac remodeling (Tokuyama and Yanagi, 2023).

### 4.2 The role of MTFP1 in regulating calcium homeostasis and myocardial contraction

Mitochondrial division is a highly regulated process that is essential for the distribution of mitochondria during cell division, the clearance of damaged mitochondria, and the overall maintenance of mitochondrial function (Vásquez-Trincado et al., 2016). An important regulator of mitochondrial division is Drp1, mitochondria accomplish their dynamic fission process through constriction and cleavage. The activity of Drp1, as well as the rate of mitochondrial division, are subject to regulation by various post-translational modifications, including phosphorylation, ubiquitination, and SUMOylation (Song and Dorn, 2015). Calcium ions are imperative for a multitude of cellular processes, including signal transduction, energy metabolism, and apoptosis (Bhandari et al., 2015). Mitochondria play a pivotal role in Ca^2+^ buffering and homeostasis, functioning as a reservoir for regulating intracellular Ca^2+^ levels. The mitochondrial uptake of Ca^2+^ is predominantly facilitated by the mitochondrial calcium uniporter (MCU), which is meticulously regulated to ensure an appropriate response to cellular signals (Leduc-Gaudet et al., 2021). Notably, mitochondria–endoplasmic reticulum contact sites (MAMs) not only regulate calcium and lipid exchange but also spatially coordinate mitochondrial fission. These sites frequently colocalize with actin filament assembly at prospective division sites, providing a structural scaffold that facilitates recruitment of the fission machinery. In particular, the actin cytoskeleton at MAMs assists in the initial constriction of the mitochondrial membrane, while dynamin-2 (DNM2) mediates the final scission step, completing the physical separation of mitochondrial segments. This coordinated interplay between MAM-associated actin dynamics and DNM2-dependent membrane severing ensures spatial precision and efficiency in mitochondrial division, thereby influencing downstream processes such as mitophagy.

The relationship between mitochondrial division and Ca^2+^ regulation is bidirectional and mutually reinforcing (Alevriadou et al., 2021). Studies have shown that increased intracellular Ca^2+^ levels can promote mitochondrial division by activating calcium-dependent phosphatases such as calcineurin. Activated calcineurin dephosphorylates Drp1 at specific serine residues (notably Ser637) located within its GTPase effector domain, a modification that occurs predominantly in the cytosol. This dephosphorylation induces a conformational change that activates Drp1 and facilitates its translocation from the cytosol to the outer mitochondrial membrane, where it assembles into oligomeric complexes to mediate membrane constriction and division (Bravo-Sagua et al., 2017). This Ca^2+^ -induced division plays a key role in promoting the mitochondrial response to cellular stress and enables the isolation and degradation of damaged mitochondria through mitophagy (Abudureyimu et al., 2024). On the other hand, the mitochondrial division process itself also influences Ca^2+^ signaling. Redistribution of mitochondria during division can alter the spatial dynamics of Ca^2+^ signaling, particularly at MAMs, where close contact between mitochondria and the endoplasmic reticulum (ER) promotes Ca^2+^ transfer (Song et al., 2023). By modulating the extent and frequency of these contacts, mitochondrial division can fine-tune Ca^2+^ signaling, which can influence processes such as energy production and apoptosis (Abudureyimu et al., 2023). Calcium ions play a crucial role in the contraction and relaxation of cardiomyocytes, and MTFP1 is involved in the homeostatic regulation of calcium ions by regulating the formation of MAMs with contact sites between mitochondria and endoplasmic reticulum (Peng et al., 2020). As a hub of intracellular storage and release of calcium ions, MAMs play a role as regulators in the rapid response needs of cardiomyocytes (Gӧbel et al., 2020). MTFP1 not only maintains the structural integrity of MAMs but also influences the electrophysiological function of the myocardium by regulating the transmembrane transport of calcium ions (Bode et al., 2021). When MTFP1 function is impaired, the structural instability of MAMs leads to disruption of calcium signaling, thereby weakening myocardial contractility, a mechanism particularly evident in myocardial hypertrophy and myocardial lesions, see Figure 4E.

### 4.3 The dual role of MTFP1 in the cardiac stress response: protector and destroyer

When cardiomyocytes face stress such as ischemia-reperfusion injury, the dysfunction of MTFP1 often exacerbates the damage of cells (Borkar et al., 2023). This is because mitochondrial fragmentation triggered by MTFP1 dysregulation leads to excess ROS production, and the accumulation of ROS further disrupts the mitochondrial membrane, leading to apoptosis and necrosis (Li et al., 2022b). However, MTFP1 acts as a cytoprotector under normal conditions by maintaining mitochondrial fusion and balancing ROS production. Studies have shown that restoring MTFP1 function can significantly reduce the apoptosis rate of cardiomyocytes under stress conditions and improve cardiac function. Therefore, the dual role of MTFP1 in the stress response makes it a key regulator of cell fate (Grumbach and Nguyen, 2019). In addition, Masahiro Morita demonstrated the nutrient-sensing mTORC1/mammalian target that stimulates the translation of MTFP1 to control mitochondrial fission and apoptosis, thereby addressing the stress response of cellular energy storm (Lozhkin et al., 2022). Expression of MTFP1 is coupled to mitochondrial recruitment of pro-fissional phosphorylation and fission-fission GTPase DRP (Wu et al., 2022b). Mitochondrial hyperfusion occurs due to reduced translation of MTFP1, which is mediated by the translation initiation factor 4E (eIF4E)-binding protein (4E-BP). Following mTOR inhibition, MTFP1 levels are uncoupled from the mTORC1/4E-BP pathway, blocking the hyperfusion response and leading to apoptosis by converting mTOR inhibitor effects from cytosuppression to cytotoxicity (He et al., 2022). These data provide direct evidence for cell survival after inhibition of mTOR by mitochondrial hyperfusion, using MTFP1 as a key effector of mTORC1 to control cell fate decisions (He et al., 2022).

### 4.4 Mechanisms of action and therapeutic potential of MTFP1 in metabolic diseases

The close association between metabolic diseases, such as diabetes, obesity, and non-alcoholic fatty liver disease (NAFLD), and mitochondrial dysfunction has been widely recognized (Liu et al., 2020). MTFP1, a key regulator in the mitochondrial fusion process, plays a central role in maintaining mitochondrial structural integrity and energy metabolic homeostasis (Alevriadou et al., 2021; Bhandari et al., 2015). Damage to mitochondrial fusion can lead to mitochondrial fragmentation, decreased energy generation efficiency, increased lipid peroxidation, and the onset of metabolic abnormalities, including insulin resistance. Studies have shown that the loss of MTFP1 function is closely associated with mitochondrial fragmentation and lipid metabolism dysregulation, thus accelerating the progression of metabolic diseases (Song et al., 2023). Activation of MTFP1 through gene therapy or small-molecule agonists has been shown to effectively improve insulin sensitivity, regulate lipid metabolism, and reduce blood glucose levels in animal models, thereby restoring mitochondrial dynamics and slowing disease progression (Wu et al., 2017). Although these preclinical studies provide strong support for its clinical application, future drug development must consider the tissue specificity of MTFP1 and its potential systemic side effects. Furthermore, excessive activation of MTFP1 may lead to increased oxidative stress, highlighting the need for caution in the precise modulation of its function to achieve a balance between therapeutic efficacy and safety (He et al., 2024; Wu et al., 2024).

## 5 The complex role of MTFP1 in cancer: multi-level regulation of mitochondrial dynamics and tumor biology

As a key GTPase on the inner mitochondrial membrane, MTFP1 plays a multi-level regulatory role in mitochondrial fusion, metabolic reprogramming, cell fate determination, and tumor progression (Tábara et al., 2024). Recent studies have shown that MTFP1 is not only involved in maintaining the structural and functional stability of mitochondria, but also profoundly affects the occurrence, development, and response to treatment by regulating the complex interactions between mitochondria and other cellular structures (Donnarumma et al., 2022). The research on targeted precision therapy for the mechanism of MTFP1 in cancer provides a new perspective for understanding the complexity of tumor biology and developing more effective cancer treatment strategies.

### 5.1 Mitochondrial dynamics and tumor metabolic reprogramming regulation of MTFP1 in cancer cells

**FIGURE 5:**
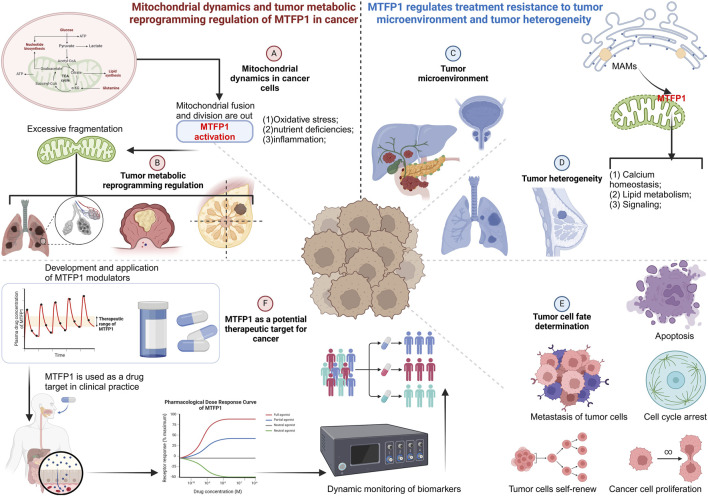
MTFP1 and cancer. **(A)** Mitochondrial dynamics of MTFP1 in cancer cells. **(B)** MTFP1 regulates tumor metabolic reprogramming. **(C)** MTFP1 and microenvironment. **(D)** MTFP1 and tumor cell fate determination. **(E)** MTFP1 as a potential therapeutic target. Abbreviations: SMURF1, Smad Ubiquitin Ligase E3; MUL1, Mitochondrial E3 Ubiquitin Protein Ligase 1; GP78, VCP Interacting Membrane Protein; NLRP3, NOD-like Receptor Family Pyrin Domain Containing 3; DAMPs, Damage-Associated Molecular Patterns.

Metabolic reprogramming of cancer cells is a core feature of tumor growth and survival, and the regulatory role of MTFP1 in this process is crucial. MTFP1 maintains the integrity of the mitochondrial network and supports oxidative phosphorylation of cells by mediating the balance of mitochondrial fusion and division (Patitucci et al., 2023). However, in the tumor microenvironment, stresses such as oxidative stress and nutrient deprivation promote tumor cells to tend to the glycolytic pathway, and mitochondrial fragmentation caused by MTFP1 dysfunction further accelerates this metabolic shift, enhancing the survival and proliferation of cancer cells (Xiao et al., 2020). In breast cancer, the abnormally high expression of MTFP1 promotes excessive mitochondrial division, weakens mitochondrial OXPHOS function, and promotes the transition of cancer cells to glycolytic metabolism (Dong et al., 2024). This transformation not only enhances the energy demand of breast cancer cells, but also changes the acid-base balance in the tumor microenvironment by accelerating the accumulation of lactate, which significantly improves the invasion and metastasis ability of cancer cells (Singh et al., 2024). Studies have shown that the aberrant expression of MTFP1 in HER2-positive breast cancer is closely related to aggressiveness, which may affect the therapeutic sensitivity of cancer cells by regulating metabolic reprogramming (Baek et al., 2023). In colorectal cancer, MTFP1 plays a key role in tumor progression by regulating mitochondrial morphology and maintaining cellular oxidative phosphorylation. Dysregulation of MTFP1 in colorectal cancer cells is strongly associated with mitochondrial fragmentation, leading to preferential use of glycolytic pathways (Haque et al., 2024). This metabolic bias not only helps tumor cells survive in a hypoxic environment, but also increases the energy supply and biosynthesis pathways of cancer cells, driving the rapid growth and invasion of tumors. In small cell lung cancer and non-small cell lung cancer (NSCLC), MTFP1 regulates the imbalance between mitochondrial fusion and division and promotes glycolytic metabolism of tumor cells (Han et al., 2023a). Its dysregulation leads to severe changes in mitochondrial morphology, which prevents cells from utilizing oxidative phosphorylation capacity, which in turn promotes the activation of aerobic glycolysis (Schneegans et al., 2024). This metabolic reprogramming provides tumor cells with the energy they need to proliferate rapidly, and there is evidence that MTFP1 is strongly associated with chemoresistance in lung cancer patients, see Figures 5A,B.

### 5.2 MTFP1 regulates the tumor microenvironment and tumor heterogeneity in treatment resistance

The tumor microenvironment (TME) plays a crucial role in tumorigenesis and progression. MTFP1 affects calcium homeostasis, lipid metabolism, and signaling by regulating the formation of mitochondria-endoplasmic reticulum contact sites MAMs, which form a complex interaction between cancer cells and the microenvironment (Kuo et al., 2022). The abnormal function of MTFP1 not only changes the proliferation and invasion ability of tumor cells, but also affects the polarization and function of tumor-associated immune cells, thereby enhancing the immune escape ability of cancer cells (Missiroli et al., 2023). In prostate cancer, MTFP1 affects calcium ion transmission and metabolic signaling by regulating the formation of MAMs. Its dysfunction leads to an imbalance of calcium ion homeostasis in prostate cancer cells, activating specific metabolic pathways that promote tumor cell proliferation (Papachristodoulou et al., 2021). The alteration of calcium ion signaling also affects the polarization of immune cells in TME, especially the transformation of macrophages to M2 type, which enhances the immune escape ability of tumors. In addition, MTFP1 promotes aggressiveness and treatment tolerance in prostate cancer by regulating lipid metabolism. Studies in hepatocellular carcinoma (HCC) have shown that MTFP1 regulates immune cell function in the tumor microenvironment by affecting calcium homeostasis and mitochondrial division of liver tumor cells (Yang et al., 2024). Its dysfunction may promote the polarization of tumor-associated macrophages, inhibit the anti-tumor immune response, and enhance the immune escape and treatment resistance of tumor cells (Juliana et al., 2020; Mima, 2022). The high expression of MTFP1 is closely related to the poor prognosis of liver cancer patients, and may be used as a potential therapeutic target. In tumor heterogeneity and treatment resistance, the energy regulation mechanism of MTFP1 in tumor cells has been gradually explored (Mamouni et al., 2021). The complex role of MTFP1 in tumor heterogeneity has been extensively studied in multiple cancers such as breast, lung, and prostate. The expression level and functional status of MTFP1 were significantly different among different tumor types and subtypes, which directly affected the sensitivity of tumor cells to chemotherapy, radiotherapy and other treatments (Ji et al., 2024). For example, in HER2-positive breast cancer, low expression of MTFP1 is associated with resistance to chemotherapeutic drugs; In non-small cell lung cancer, the dysfunction of MTFP1 leads to the cancer cells' resistance to radiotherapy (Meng et al., 2024). An in-depth understanding of the differences in MTFP1 expression may provide a new direction for future precision medicine, as shown in Figures 5C,D.

### 5.3 Relationship between MTFP1 and tumor cell fate determination

By regulating mitochondrial division and fusion, MTFP1 determines the fate of cells under stressful conditions, especially the balance between apoptosis and autophagy. Its dysfunction is often associated with mitochondrial dysfunction, which promotes cancer cell resistance to apoptotic signals and further promotes the malignant progression of tumors (Deng and Haynes, 2017). In breast cancer, MTFP1 maintains the balance between apoptosis and autophagy by regulating mitochondrial structure. Its aberrant expression weakens the activity of the apoptotic pathway, promotes the survival of cancer cells under stress conditions, and enhances the resistance to anticancer drugs (Zuo et al., 2022). This mechanism provides a survival advantage to cancer cells through the autophagy pathway, allowing them to gain more resources to support proliferation in the harsh tumor microenvironment. In prostate cancer, MTFP1 affects the proliferative ability of cancer cells by regulating cell cycle-related signaling pathways (He et al., 2023). Its dysregulated expression leads to mitochondrial dysfunction and activates signaling pathways that promote cell proliferation, further promoting the rapid proliferation of cancer cells. This regulatory effect of MTFP1 is particularly prominent in the progression of prostate cancer and is closely related to poor prognosis, as shown in Figure 5E.

### 5.4 MTFP1 as a potential therapeutic target for cancer

MTFP1 is considered a potential target in cancer therapy due to its critical role in a variety of cancers. Targeting the regulatory mechanism of MTFP1 may restore the normal function of mitochondria, reverse metabolic reprogramming, and inhibit the proliferation and survival of cancer cells (Pokharel et al., 2023). The following are targeted strategies for different cancers: (1) Targeted therapy for breast cancer, abnormal expression of MTFP1 in HER2-positive and triple-negative breast cancer is related to treatment sensitivity. Therapies targeting MTFP1 may enhance the sensitivity of cancer cells to treatment by restoring mitochondrial function (maintain cellular energy production, regulate apoptosis, and balance cellular stress responses) and decreasing the activity of glycolytic metabolism (Rolver et al., 2024). Drug development targeting the pathway related to MTFP1 and mitochondrial dynamics is expected to significantly improve the therapeutic effect in breast cancer patients. (2) Combined targeted therapy in lung cancer: In non-small cell lung cancer, the combined targeted therapy of MTFP1 and mitochondrial dynamics-related proteins (such as Drp1, OPA1, etc.) has shown significant anti-tumor effects. By simultaneously regulating the mitochondrial division and fusion pathways, combined therapy targeting MTFP1 and related proteins can effectively inhibit the metabolic reprogramming of cancer cells and increase the efficacy of chemotherapy or radiotherapy (Li et al., 2022a). (3) Prognostic markers in colorectal cancer.

Aberrant expression of MTFP1 in colorectal cancer can serve as a potential prognostic marker. By detecting the expression level of MTFP1, the investigator can predict the patient’s sensitivity to treatment and develop a personalized treatment plan (Wu et al., 2024; Pan et al., 2024). In the future, targeted therapy targeting MTFP1 may provide more precise treatment options for colorectal cancer patients, as shown in Figure 5F. Given the multiple roles of MTFP1 in cancer, targeting the function of MTFP1 may provide a new strategy for cancer treatment. For example, reconstructing the normal fusion state of mitochondria by restoring the function of MTFP1 may effectively inhibit metabolic reprogramming of tumor cells and enhance their sensitivity to treatment (Srinivasan et al., 2017; He et al., 2024). In addition, MTFP1 can also be used as a biomarker to predict treatment response and patient prognosis for different tumor subtypes. In future studies, in-depth exploration of the specific mechanisms of MTFP1 in different tumor types will help to develop more precise and effective treatments (Maycotte et al., 2017).

## 6 MTFP1 and muscle health

As a key GTPase located in the mitochondrial inner membrane, MTFP1 plays a crucial role in regulating mitochondrial dynamics, particularly in skeletal muscle and cardiac systems. Its functions encompass metabolic homeostasis, muscle fiber type regulation, exercise adaptation, and muscle protective functions under pathological conditions (Quiles and Gustafsson, 2022). Skeletal muscle and the heart, both highly dependent on mitochondrial function, are responsible for maintaining cellular energy production, regulating apoptosis, and balancing cellular stress responses. The health of these tissues requires not only a stable mitochondrial network but also the dynamic coordination of complex signaling pathways (Leduc-Gaudet et al., 2021). The specific mechanisms by which MTFP1 operates in these systems will be further discussed below.

### 6.1 The core role of MTFP1 in maintaining mitochondrial dynamics in skeletal muscle and cardiac smooth muscle

**FIGURE 6:**
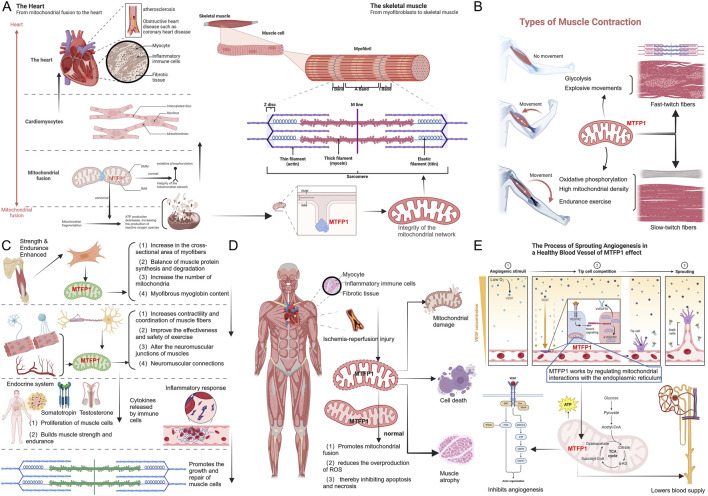
Mechanism of action of MTFP1 on myocardial and skeletal muscles. **(A)** Effect of excessive mitochondrial division under the influence of MTFP1 on cardiomyocytes and vascular smooth muscle. **(B)** The role of mitochondria on skeletal muscle under the influence of MTFP1. **(C)** Role of MTFP1 in muscle adaptation and exercise endurance. **(D)** Effects of MTFP1 on cells in physiological and pathological states. **(E)** Role of MTFP1 in angiogenesis and metabolic adaptation of skeletal muscle. Abbreviations: ATP, Adenosine Triphosphate; TCA, Tricarboxylic Acid Cycle; α-KG, Alpha-Ketoglutarate; OMM, Outer Mitochondrial Membrane; IMM, Inner Mitochondrial Membrane; SRC, Src Proto-Oncogene, Non-Receptor Tyrosine Kinase; VEGF, Vascular Endothelial Growth Factor; SCK, Src-Related Kinase; VRAP, Vacuolar ATPase Assembly Protein; PI3k, Phosphoinositide 3-Kinase; MKK, Mitogen-Activated Protein Kinase Kinase; MAPK, Mitogen-Activated Protein Kinase; HSP27, Heat Shock Protein 27.

The high-energy demand of skeletal muscle and cardiac tissue makes mitochondrial dynamics a core aspect of cellular physiological activity. MTFP1 maintains mitochondrial network integrity by promoting mitochondrial fusion and supports oxidative phosphorylation within the cell (Chan, 2020; Shi et al., 2024). Mitochondrial fusion optimizes the assembly of respiratory chain complexes, thereby enhancing mitochondrial energy production efficiency. When MTFP1 function is impaired, mitochondrial fragmentation occurs, leading to a decrease in ATP output, an increase in reactive oxygen species (ROS) generation, and consequently, oxidative stress accumulation that triggers muscle cell dysfunction (Ihenacho et al., 2021). Fragmented mitochondria are unable to efficiently repair damaged DNA, further exacerbating metabolic disturbances, ultimately resulting in declines in skeletal muscle and cardiac function. This dynamic imbalance is closely associated with aging, muscle atrophy, and metabolic diseases (Kubat et al., 2023). Studies have shown that in the heart, mitochondrial deletion of MTFP1 results in specific inner membrane defects, including ineffective proton leakage of adenine nucleotide translocases and heightened sensitivity to mitochondrial permeability transition pores (Ihenacho et al., 2021; Donnarumma et al., 2022). Additionally, MTFP1 participates in these processes through both physical and genetic interactions, unveiling its new role in controlling bioenergetics and cellular death sensitivity. Research by the Wang team has revealed the role of MIR4435-2HG in cardiac ischemic injury and cardiomyocyte apoptosis, unveiling a novel MIR4435-2HG/miR-125a-5p regulatory axis in cardiac ischemia/reperfusion injury, providing potential therapeutic targets for ischemia/reperfusion-induced myocardial damage (Wang et al., 2022), as shown in Figure 6A.

### 6.2 Role of MTFP1 in the differentiation and metabolic regulation of skeletal muscle fiber types

Skeletal muscle is composed of a variety of fiber types, including type I slow-twitch fibers and type II fast-twitch fibers, which differ significantly in function and metabolic requirements. Type I slow-twitch fibers are oxidative phosphorylation dependent and have high mitochondrial density, making them suitable for endurance exercise (Rahman et al., 2024); Type II fast-twitch fibers are more dependent on glycolysis and are suitable for explosive movements. MTFP1 affects the differentiation of myofibers by regulating the mitochondrial fusion state (Castro-Sepulveda et al., 2023). In type I muscle fibers, high expression of MTFP1 helps maintain the integrity of the mitochondrial network, supporting sustained energy supply primarily through oxidative metabolism. In contrast, in type II muscle fibers, MTFP1 may adapt to their rapid energy demands driven by glycolysis by regulating mitochondrial morphology and function, demonstrating its functional diversity under different metabolic contexts. In type I muscle fibers, high expression of MTFP1 helps maintain the integrity of the mitochondrial network, supporting sustained energy supply primarily through oxidative metabolism. In contrast, in type II muscle fibers, MTFP1 may adapt to their rapid energy demands driven by glycolysis by regulating mitochondrial morphology and function, demonstrating its functional diversity under different metabolic contexts (Mima, 2022). Dysregulation of MTFP1 can lead to abnormal fiber type switching, which in turn impairs the muscles' ability to adapt to external loads and metabolic demands (Yasuda et al., 2023). Studies have shown that the role of MTFP1 in muscle adaptation is important in preventing muscle degeneration and imbalance of myofiber types in aging and disease states (Cui et al., 2024), see Figure 6B.

### 6.3 Role of MTFP1 in muscle adaptation and exercise endurance

Exercise-induced mitochondrial biogenesis and adaptive changes in skeletal muscle depend on mitochondrial homeostasis. MTFP1 enhances exercise tolerance by enhancing mitochondrial fusion, improving oxidative phosphorylation efficiency, and optimizing the metabolic function of muscle cells. During exercise, MTFP1 expression is upregulated, allowing muscles to effectively adapt to high-intensity and long-term energy demands (Axelrod et al., 2019). Especially after endurance exercise, the continuous upregulation of MTFP1 helps mitochondrial repair and regeneration, and improves muscle recovery after exercise. MTFP1 is closely related to post-exercise mitochondrial remodeling and functional enhancement, which means that it plays a crucial role in regulating the long-term adaptation of skeletal muscles to exercise and the improvement of exercise performance (Campos et al., 2023). MTFP1 is associated with myocardial energy metabolism adaptation and cardiac exercise tolerance, therefore, the regulation of Mtfp1 expression may be a novel treatment for chemotherapy-induced cardiotoxicity, as shown in Figure 6C.

### 6.4 Protective effect of MTFP1 in muscle pathological states

MTFP1 plays a key protective role by maintaining mitochondrial functional integrity in a variety of muscle pathological states, especially in the presence of ischemia-reperfusion injury or metabolic disorders (Hong et al., 2022). The increase in mitochondrial stress due to ischemia-reperfusion is often accompanied by excessive mitochondrial division, and MTFP1 inhibits apoptosis and necrosis by promoting mitochondrial fusion and reducing the overproduction of ROS. Loss of function of MTFP1 exacerbates mitochondrial damage, which in turn accelerates cell death, muscle atrophy, and loss of function (Morita et al., 2017). In the pathological state, enhancing the function of MTFP1 can effectively reduce mitochondrial damage, maintain cell viability and metabolic homeostasis, so MTFP1 is considered a potential therapeutic target, especially in the intervention of muscle degenerative diseases (Cai et al., 2024). Professor Cui performed bioinformatics analysis of common differentially expressed genes (cDEG), fasting differential genes (fDEGs), and mitochondria-related genes, validated the overlapping genes identified by RT-qPCR and Western blotting in various mouse models of sarcopenia, and identified three hub genes (Acss1, Mtfp1, and Oxct1), which are closely associated with mitochondrial dysfunction in sarcopenia (Cui et al., 2024), See Figure 6D.

### 6.5 Role of MTFP1 in angiogenesis and metabolic adaptation of skeletal muscle

Skeletal muscle not only requires strong metabolic function, but also relies on adequate blood supply and angiogenesis. MTFP1 influences angiogenesis processes within skeletal muscle by regulating mitochondrial interactions with endoplasmic reticulum. Under exercise or pathological conditions, MTFP1 can promote angiogenesis and maintain blood supply by activating metabolic regulatory pathways such as PGC-1α (You et al., 2024). Loss of MTFP1 not only inhibits angiogenesis and reduces blood supply, but also leads to dysregulation of muscle metabolism, which in turn impairs muscle endurance and adaptability. Studies have shown that the regulatory relationship between MTFP1 and angiogenesis suggests that MTFP1 plays an important role in maintaining muscle metabolic homeostasis and functional recovery in sports medicine and pathological conditions (Sollanek et al., 2017). Wang et al. regulate mitochondrial fission and apoptosis in the heart by directly targeting and downregulating miR-652-3p (Wang et al., 2017a); This, in turn, blocks mitochondrial fission and cardiomyocyte death by inhibiting MTP18 translation. MTP18 deficiency reduces mitochondrial fission and inhibits cardiomyocyte apoptosis and Myocardial infarction (MI). miR-652-3p directly downregulates MTP18 and attenuates mitochondrial fission, cardiomyocyte apoptosis, and MI *in vitro* and *in vivo* (Wang et al., 2017a), see Figure 6E.

MTFP1 is involved in the functional regulation of skeletal muscle through multi-level mechanisms, especially in mitochondrial dynamics, fibrous differentiation, metabolic adaptation, and protection under pathological conditions (Ahuja et al., 2022). The dysregulation of MTFP1 is closely related to a variety of muscle dysfunction and metabolic diseases, and in-depth study of its mechanism of action is not only helpful to understand the complexity of muscle physiology and pathological processes, but also provides potential targets and strategies for the treatment of muscle-related diseases (Dulac et al., 2021).

## 7 Frontiers and challenges of targeting MTFP1 in precision medicine

As a central regulatory factor in the mitochondrial fusion process, MTFP1 plays a pivotal role in maintaining cellular energy metabolism, redox homeostasis, and cell fate determination. Its broad expression and functional dysregulation in multiple disease systems—including metabolic disorders, cardiovascular diseases, and cancer—highlight its unique advantage as a “cross-system therapeutic target.” Under pathological conditions, MTFP1 modulates mitochondrial dynamic equilibrium, thereby influencing the cell’s capacity to respond to environmental stress and participating in key processes of programmed cell death, such as apoptosis, autophagy, and ferroptosis (Fukuda et al., 2023). Consequently, MTFP1 has evolved from a basic mitochondrial structural maintenance protein to a critical node within the cellular fate-regulation network. This property not only bridges fundamental research and therapeutic development but also provides a theoretical foundation for cross-disease and cross-system precision medicine strategies.

Future therapeutic interventions will increasingly rely on multi-target integration and system-level regulation. Mitochondrial dynamics–related proteins closely associated with MTFP1—such as Drp1, MFN1/2, and OPA1—have been shown to cooperatively regulate mitochondrial morphology and function across various pathological models, suggesting that combination targeting may further enhance therapeutic efficacy. Against this backdrop, small-molecule agonists and inhibitors of MTFP1, as well as RNA interference and gene-editing tools, have demonstrated potential in animal studies to improve metabolic disorders and suppress tumor progression. However, clinical translation still faces substantial challenges, including tissue-specific regulation, targeting and delivery efficiency, penetration across biological barriers, dose optimization, and safety evaluation. To address these issues, future directions should focus on developing intelligent delivery platforms, optimizing molecular screening systems, and integrating structural biology approaches to design next-generation regulatory tools. Moreover, the paradigm of personalized medicine demands systematic evaluation of MTFP1 genetic polymorphisms, disease subtype correlations, and interindividual variability in therapeutic responses. By combining artificial intelligence with multi-omics big data, predictive modeling and feedback-based therapeutic monitoring could be established, paving the way for truly “tailored therapeutics.” This approach holds promise for advancing MTFP1-targeted strategies from bench to bedside, benefiting patient populations burdened by metabolic diseases, cancer, and other high-impact conditions (Table 2).

**TABLE 2:** Proteins and peptides experimentally associated with MTFP1-related mitochondrial regulation.

Compound class	Compound	Model	Main mechanism of action	Clinical therapeutic implications	References
Direct target drugs for MTFP1	STMP1	Xenograft mice	STMP1 overexpression results in mitochondrial redistribution to the leading edge of the cell, enhancing lamodipodia formation	STMP1 is a key regulator of metastasis and a novel unit of mitochondrial fission protein mechanism, providing a potential therapeutic target for the treatment of cancer metastasis	Xie et al. (2022)
	ALKBH5	Stellate cells, rat liver tissue	LKBH5 regulates the m6A modification of Drp1, promotes its translation, and enhances mitochondrial fission, cell proliferation and migration in HSCs	Modulating ALKBH5 activity to control mitochondrial fission and HSC behavior, alleviate liver fibrosis, MTFP1 provides a new target for the diagnosis and treatment of liver fibrosis	Geng et al. (2022)
	DEX	C57BL/6J mice, NR8-383 cells	DEX reduced oxidative stress index (OSI), ameliorated mitochondrial dysfunction, upregulated HIF-1α and HO-1 expression, and was associated with mitochondrial fusion	DEX treatment ameliorates endotoxin-induced acute lung injury by modulating the HIF-1a/HO-1 signaling pathway to maintain the homeostasis of mitochondrial fusion/fission	Shi et al. (2021)
	Beta-amyloid protein (Aβ) overphosphorylates tau (P-Tau)	Cardiomyocytes	Aβ and P-Tau decrease autophagy and mitochondrial autophagy protein levels (Drp1/PINK1/parkin), resulting in damaged mitochondria and other cellular debris that cannot be effectively cleared in AD neurons	Understanding the mechanisms by which Aβ and P-Tau lead to autophagy and mitophagy defects in Alzheimer’s disease can help develop new therapeutic strategies to improve neuronal function and delay disease progression	Reddy and Oliver (2019)
	MTP18/MTFP1	Retinal ganglion cells (RGCs)	MTFP1 (MTP18) is essential for maintaining mitochondrial size and volume. Knockdown of MTP18 promoted axon outgrowth, suggesting that MTP18 expression was regulated by the transcription factors Kruppel-like factor (KLF)-7 and −9	The role of MTP18 in axonal regeneration signaling, and to study the therapeutic effectiveness of MTP18 expression inhibition in axonal degenerative events in the central nervous system (CNS)	Kreymerman et al. (2019)
	miR-125b-5p	Endometrial cancer cells	miR-125b-5p targets the putative binding site on MTFP1 3′UTR to reduce MTFP1 expression and inhibit cellular malignant behavior	miR-125b-5p inhibits the EC cell malignant phenotype by targeting MTFP1, laying the foundation for the clinical treatment of endometrial cancer	Pan et al. (2023)
	Tumor suppressor p53	Tumor cells	p53 inhibits the target of mammalian rapamycin complex 1 (mTORC1) signaling to attenuate protein levels of mitochondrial fission process 1 (MTFP1) and promote fission-promoting dynein-related protein 1 (Drp1) phosphorylation	A novel mitochondria-dependent molecular mechanism was revealed, i.e., the basis of the metastatic phenotype of p53-damaged cancer was associated with MTFP1	Phan et al. (2022)
	Suvorexant	Stress-restressed rats with PTSD-like symptoms	SRS exposure leads to increased mitochondrial fission and decreased fusion, i.e., excessive increase in MTFP1, and activation of the mTOR pathway causes PTSD-like symptoms, while the combination of suvorexant and mTOR can improve mitochondrial dynamics	Targeting orexinergic and mTOR pathways may have beneficial synergistic effects in the treatment of PTSD	Prajapati et al. (2024)
	Small molecule echinacoside (ECH)	Middle cerebral artery occlusion mice (MCAO)	CH allosterically regulates the conformation of CK2α′ to recruit the basic transcription factor 3 (BTF3) to form a bivalent protein complex. Then, the CK2α'/BTF3 complex promotes the nuclear translocation of β-catenin to activate the TCF/LEF transcription factor and stimulate the transcription of mitochondrial fusion gene Mfn2	Reveals that CK2 is essential for promoting mitochondrial fusion in a Wnt/β-catenin-dependent manner, and suggests pharmacologically targeted CK2 for the treatment of ischemic stroke treatment strategies, and is associated with an aberrant increase in gene hypertranscriptional grade MTFP1 for MFN	Zeng et al. (2021)
	QSYQ	Myocardial infarction rats	QSYQ promotes mitochondrial biogenesis (PGC-1α, Nrf1 and TFAM) and mitochondrial fusion (MFN-2 and OPA1) and inhibits phosphorylation of mitochondrial hyperdivision (Drp1) at ser616 *in vitro* and *in vivo*, suggesting that the cardioprotection of QSYQ is related to the promotion of mitochondrial biogenesis and homeostasis	QSYQ improves mitochondrial biogenesis and homeostasis to alleviate MI-induced ferroptosis	Wu et al. (2023)
	GTPBP8	Cell	Depletion of GTPBP8 leads to dramatic elongation and interconnectivity of mitochondria. Overexpression of GTPBP8 shifts mitochondrial morphology from tubular to fragmented	This study reveals the critical role of GTPBP8 in mitochondrial fission and its interrelationship with Drp1, providing potential targets and biomarkers for the treatment of mitochondrial dysfunction-related diseases	He et al. (2024)
Indirect target drugs for MTFP1	Ilexgenin A (IA)	Atherosclerotic mice	IA promotes PSMB5 expression and activates Nrf2 signaling pathway, inhibits Drp1 expression and mitochondrial fission, improves endothelial dysfunction, reduces inflammation and oxidative stress, and exerts anti-atherosclerosis effects through MTFP1	IA promotes PSMB5 expression in an Nrf2-dependent manner, inhibits mitochondrial fission, and ameliorates endothelial dysfunction. It lays the foundation for the future development of IA as a drug for the prevention and treatment of AS	Zhu et al. (2019)
	Gypenosides (GYP)	H9c2 cardiomyocytes	rescue defective mitophagy to exert cardioprotective effects, and PI3K/Akt/GSK-3β/Mcl-1 signaling may be involved in its process	Exert cardioprotective effects in cardiomyocytes by activating the PI3K/Akt/GSK-3β/Mcl-1 signaling pathway to rescue mitophagy, which provides a basis for their potential application in the treatment of heart failure (HF)	Zheng et al. (2024)
	Korean Red Ginseng (KRG)	Testosterone propionate (TP)-induced BPH and TP-treated LNCaP cells	KRG inhibits mitochondrial fission by enhancing DRP-1 (ser637) phosphorylation, thereby reducing cell proliferation and promoting apoptosis, effectively alleviating BPH symptoms	KRG provides a promising clinical approach for the treatment of benign prostatic hyperplasia (BPH) by regulating mitochondrial dynamics, inhibiting cell proliferation and promoting apoptosis	Hong et al. (2024)
	Neuraminidase 1	DOX-induced cardiomyopathy rats	DOX enhances Drp1-dependent mitochondrial fission and PINK1/Parkin-mediated mitophagy by upregulating NEU1 expression, leading to cardiomyocyte apoptosis and cardiac impairment, while OSE exerts cardioprotective effects by inhibiting this pathway	NEU1 plays a key role in DOX-induced cardiomyopathy, while the NEU1 inhibitor oseltamivir (OSE) exhibits potential cardioprotective effects by inhibiting Drp1-dependent mitochondrial fission and mitophagy, which may provide a new clinical strategy for the prevention or treatment of anthracycline-induced cardiotoxicity	Qin et al. (2021)
	SNO-Drp1	Mice	Inhibition of MAP4K4 mitigates cardiac insufficiency by promoting S-nitrosylation of Drp1 by inhibiting GPX4 expression, leading to mitochondrial dysfunction and cardiac microvascular disease, thereby exacerbating cardiac insufficiency	MAP4K4 provides a new potential target for the treatment of diabetic cardiomyopathy by promoting cardiac microvascular dysfunction and cardiac insufficiency caused by S-nitrosylation of Drp1	Chen et al. (2024)
	ferulic acid	Rats were fed high-fat/high-fructose/streptozotocin	Under high glucose conditions, the distortion of MAM (mitochondria-associated endoplasmic reticulum membrane) and mitochondrial dysfunction lead to the occurrence and development of diabetic cardiomyopathy, and ferulic acid (FeA) can effectively reverse these pathological changes	Ferulic acid may be an effective supplement for the treatment of diabetic cardiomyopathy by modulating MAM	Salin Raj et al. (2023)
	Copper (ii) complexes	H9c2 cardiomyocytes	The copper (ii) complex induces a mechanism of apoptosis leading to DNA damage and mitochondrial dysfunction, suggesting that it has potential antitumor effects, especially in cisplatin-resistant cells	Studies have found that copper (ii) complexes can enhance apoptosis by inducing DNA damage and mitochondrial dysfunction, showing potential application value in anti-tumor therapy	Zerihun and Qvit (2023)
	BC1618	High-fat diet-induced obesity in mice	BC1618 inhibits the action of the ubiquitin E3 ligase subunit protein Fbxo48, indirectly causes an excessive increase of MTFP1, prevents the polyubiquitination and proteasomal degradation of active phosphorylated Ampkα (pAmpkα), and improves the biological activity of Ampk	BC1618 enhances Ampk activity, promotes mitochondrial fission, autophagy, and improves hepatic insulin sensitivity, which has potential application value in the treatment of obesity-related metabolic disorders	Liu et al. (2021b)
	Resveratrol	High-fat (HF) diet is fed 15-week susceptible mice with accelerated senescence	Resveratrol inhibits mitochondrial dynamics, reduces inflammation and activates the Wnt/β-catenin signaling pathway, preventing cognitive impairment and aging-related neurodegenerative diseases caused by high-fat diets	Resveratrol may be a potential effective drug for the prevention or treatment of aging and neurodegenerative diseases, and the mechanism is related to MTFP1	Ludwig-Słomczyńska et al. (2024)

### 7.1 Regulation of MTFP1 in metabolic diseases: potential from basic biology to clinical applications

The close relationship between metabolic diseases such as diabetes, obesity, and fatty liver disease and mitochondrial dysfunction has been extensively studied and confirmed (Morita et al., 2017; Zhou et al., 2022). The potential of MTFP1 in these diseases stems first from its regulation of mitochondrial fusion processes. When mitochondrial fusion is impaired, cells are unable to effectively maintain the integrity of the mitochondrial network, resulting in decreased energy productivity, increased oxidative stress, and metabolic dysfunction (Yu et al., 2023). By targeting the function of MTFP1, mitochondrial homeostasis can be restored and the progression of metabolic diseases can be mitigated. In recent years, several studies have shown that gene therapy or small molecule activators of MTFP1 effectively improve insulin resistance and lipid metabolism disorders in animal models, which lays the foundation for future clinical applications (Grumbach and Nguyen, 2019; Haque et al., 2024). However, the functional specificity of MTFP1 in different tissues requires that we must consider tissue specificity and systemic side effects when developing targeted drugs (Markovinovic et al., 2022; Johnson et al., 2021).

### 7.2 The complex role of MTFP1 in cancer: from metabolic reprogramming to cell fate determination

A key feature of cancer cells is the reprogramming of their metabolism and the enhancement of their anti-apoptotic capacity, which are largely dependent on aberrant regulation of mitochondrial dynamics (Cisneros et al., 2022). MTFP1 affects metabolic pathways, apoptosis signaling, and cell cycle regulation in cancer cells by regulating mitochondrial fusion. The dysregulation of MTFP1 in cancer cells is often manifested by increased mitochondrial fragmentation, a phenomenon that is not only related to the Warburg effect (cancer cells favor the glycolytic pathway under aerobic conditions), but also enhances the cell’s resistance to apoptosis (Lin and Beal, 2006; Romani et al., 2022). Drug development targeting MTFP1, especially by inhibiting MTFP1-induced mitochondrial fragmentation, can impair the metabolic adaptability of cancer cells and increase their sensitivity to conventional chemotherapy and radiotherapy (Yu et al., 2021). Current studies have demonstrated that inhibition of MTFP1 can effectively reduce tumor volume and inhibit metastasis, but it also presents an important challenge of how to specifically target MTFP1 dysfunction in cancer cells without damaging normal cells.

### 7.3 Future outlook of MTFP1 as a drug target: a new direction for precision medicine

Although significant progress has been made in the study of MTFP1 as a drug target, its clinical application still faces challenges. First, the functional regulation of MTFP1 involves a complex intracellular signaling network with close interactions with a variety of mitochondrial kinetic proteins, which makes it difficult for a single targeting strategy to produce the desired effect (Rocca et al., 2023). Therefore, future research should focus on the development of combination therapy strategies to achieve a more comprehensive treatment effect by simultaneously regulating multiple key nodes. Second, the functional diversity and specificity of MTFP1 in different tissues require us to fully consider the tissue selectivity and safety of targeted drugs in drug development. Finally, although the gene therapy strategy of MTFP1 has shown great potential in animal models, its safety and efficacy in humans still need to be verified through large-scale clinical trials (Rizzo et al., 2023). In the future, with the continuous progress of gene editing technology, RNA interference technology and small molecule drug screening technology, the development of MTFP1 as a drug target will gradually move towards precision and personalization. By understanding the molecular mechanisms of MTFP1 in different diseases, scientists can develop more precise treatment strategies, provide patients with more effective treatment options, and ultimately improve disease prognosis and improve patients’ quality of life (Sainero-Alcolado et al., 2022).

## 8 Prospect of clinical application of MTFP1 protein: a new era of precision medicine and mitochondrial function regulation

MTFP1 plays a central role in regulating mitochondrial dynamics and is involved in maintaining the balance between intracellular energy metabolism and signal transduction. As the central role of mitochondrial function in a variety of complex diseases has been gradually revealed in recent years, the exploration of MTFP1 as a potential therapeutic target has gradually become a hot topic (Kerr et al., 2017). From the perspective of the frontiers of cell biology, this article discusses the broad prospects of MTFP1 in clinical applications and highlights its potential role in precision medicine and the management of complex diseases.

### 8.1 Centrality of MTFP1 in mitochondrial dynamics

Mitochondria serve as the center of intracellular energy metabolism and their homeostasis is essential for maintaining cellular homeostasis. MTFP1 ensures the integrity and functionality of the mitochondrial network by regulating the mitochondrial fusion process. Mitochondrial homeostasis not only influences the production of ATP, but also plays a key role in regulating intracellular calcium balance, ROS production and transduction of apoptosis signals. Therefore, any deviation in MTFP1 function can lead to severe cellular dysfunction, which in turn can lead to the occurrence of various diseases (Cen et al., 2021).

### 8.2 Prospects for the use of MTFP1 in the treatment of metabolic diseases

Metabolic diseases such as diabetes and obesity are often associated with significant changes in mitochondrial function (maintain cellular energy production, regulate apoptosis, and balance cellular stress responses). The central role of MTFP1 in maintaining the structural and functional integrity of mitochondria makes it a potential therapeutic target for these diseases. Studies have shown that loss of function of MTFP1 is closely related to mitochondrial fragmentation, energy metabolism disorders, and lipid peroxidation. These changes not only accelerate the progression of metabolic diseases, but can also lead to increased insulin resistance. Preclinical studies have shown that mitochondrial dynamics can be effectively improved by restoring or improving the function of MTFP1, thereby reversing metabolic disorders. For example, activation of MTFP1 in mouse models of obesity and type 2 diabetes was able to significantly improve mitochondrial fusion status, restore insulin sensitivity, and reduce blood glucose levels (Prastya et al., 2020). These results suggest that MTFP1 modulators may represent a new way to treat metabolic diseases. However, the question of how to precisely modulate MTFP1 to avoid potential side effects such as the increase in oxidative stress that can be triggered by overactivation still remains an important direction for future research [108].

### 8.3 Challenges and opportunities for the use of MTFP1 in cancer therapy

Reprogramming energy metabolism in cancer cells is an important basis for their ability to rapidly proliferate and invade, and mitochondria play a central role in this process. MTFP1 regulates the metabolic flexibility and antiapoptotic capacity of cancer cells by affecting the balance of mitochondrial fusion and division. In a variety of cancers, MTFP1 dysfunction is strongly associated with tumor progression and drug resistance (Ludwig-Słomczyńska et al., 2024). Strategies targeting MTFP1 may impair the metabolic adaptation of cancer cells by affecting their mitochondrial dynamics and increasing their sensitivity to apoptosis. For example, studies have shown that inhibiting MTFP1 function can trigger mitochondrial fragmentation in cancer cells, reducing their ability to adapt to energy metabolism and thereby increasing the effects of chemotherapy and radiation therapy. However, due to the important role of MTFP1 in normal cells, this therapeutic strategy also faces challenges in terms of targeted selectivity and possible side effects. Future research should focus on developing highly specific MTFP1 inhibitors or activators in combination with other treatments such as immunotherapy to achieve more effective and safer cancer treatments (Zhao et al., 2021; Malik and Czajka, 2013).

### 8.4 Prospects for clinical application of MTFP1 and personalized medicine

With the development of genomics and personalized medicine, the clinical application prospects of MTFP1 have been further expanded. By analyzing the patient’s genetic background, the response to MTFP1-targeted therapy can be predicted to create a more accurate treatment plan. For example, polymorphisms in the MTFP1 gene can influence a patient’s sensitivity to certain modulators, and this information can help doctors choose the most appropriate treatment strategy (Park et al., 2018; Liu et al., 2024). In addition, safety monitoring of MTFP1 as a therapeutic target is also a key issue in clinical applications. Through biomarker-based dynamic monitoring, treatment dose and regimen can be adjusted in a timely manner, reducing the risk of possible side effects and improving the treatment effect. The clinical application of MTFP1 as a drug target is promising and covers a variety of complex diseases such as metabolic diseases and cancer (Duroux-Richard et al., 2016). However, the development and application of MTFP1 modulators still faces numerous challenges, including how to ensure the specificity and safety of the treatment and how to achieve precise regulation in complex biological contexts. Future research must bridge the gap between basic research and clinical applications to bring MTFP1-targeted therapies from the laboratory to the clinic and provide patients with more effective and safer treatment options.

## 9 Conclusion and outlook

MTFP1, as a core regulator of mitochondrial dynamics, plays a crucial role in maintaining cellular metabolic homeostasis, redox balance, and cell fate decisions. Extensive research has demonstrated that MTFP1 dysfunction can lead to mitochondrial fragmentation, enhanced oxidative stress, and cell death, all of which contribute to the onset and progression of metabolic diseases, cardiovascular disorders, and cancer, underscoring its potential as a therapeutic target for multi-system diseases. Despite the promising therapeutic prospects of MTFP1, its clinical translation faces numerous challenges. The tissue-specific functional heterogeneity of MTFP1 suggests that targeted modulation could trigger tissue-specific side effects, while issues of dose control and target specificity must be rigorously addressed. Currently, gene therapy and small-molecule interventions have shown positive progress in animal models, but their safety and efficacy in humans still require further validation. Looking ahead, a deeper understanding of MTFP1’s functional properties in cellular signaling pathways and specific disease contexts will provide a more solid theoretical foundation for its targeted intervention. With advancements in gene editing, targeted delivery systems, and multi-omics technologies, the development of highly selective, low-toxicity MTFP1 modulation strategies has become increasingly feasible. Integrating MTFP1 biology into the framework of precision medicine holds the promise of advancing personalized treatment strategies for mitochondrial-related diseases, ultimately improving patient prognosis and fostering breakthroughs in the integration of mitochondrial biology with clinical therapies.

**FIGURE 7:**
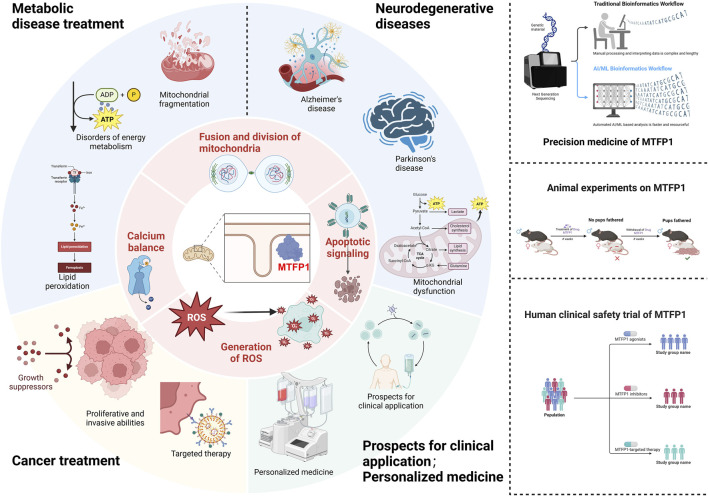
Modern applications and precise targeting of MTFP1. Abbreviations: ADP, Adenosine Diphosphate; ROS, Reactive Oxygen Species; ATP, Adenosine Triphosphate.

### Chemical compounds

Alpha-Ketoglutarate (PubChem CID:51); Adenosine Diphosphate (PubChem CID:6022); Adenosine Triphosphate (PubChem SID:5957); Guanosine Triphosphate (PubChem CID:135398633); Nicotinamide Adenine Dinucleotide, Reduced Form (PubChem CID:21604869); N-Acetylcysteine (PubChem CID:12035); Reactive Oxygen Species (PubChem SID:53787197); STMP1 (PubChem SID:491314938); ALKBH5 (PubChem CID:2943205); Biotinyl-amyloid beta-protein (PubChem CID:3200381); MTP18 (PubChem SID:85123418); IH-310394-07 (PubChem SID:251848198); Tumor suppressor p53 (PubChem SID:472419474); Suvorexant (PubChem CID:24965990); GTPBP8 (PubChem SID:134287128); Ilexgenin A (PubChem CID:21672638); Gypenosides (PubChem CID:9898279); Korean Red Ginseng (PubChem CID:505635692); Neuraminidase 1 (PubChem CID:65028); SNO-Drp1 (PubChem CID:134817168); ferulic acid (PubChem CID:45858); Copper(ii) complexes (PubChem CID:3032555); Resveratrol PubChem CID:445154).

## Glossary

α-KG: Alpha-Ketoglutarate
ADP: Adenosine Diphosphate
ANT: Adenylate translocase
ALR: Adenosine L-Ribose
AKI: Acute Kidney Injury
AKT: RAC Alpha Serine/Threonine-Protein Kinase
AMPK: AMP-activated protein kinase
ATP: Adenosine Triphosphate
BiK: Bcl-2-like protein 11
BP1: Mitochondria-Related Biomarker 1
cDEG: cavity differentially expressed genes
CMA: Chaperone-Mediated Autophagy
CypD: cyclic adenosine monophosphate-dependent phosphokinase
Cyt C: cytochrome C
DAMPs: Damage-Associated Molecular Patterns
DoX: Doxorubicin
Drp1: Dynamin-related protein 1
EPK1: Extracellular Signal-Regulated Kinase 1
ER: Endoplasmic reticulum
EC: Endothelial Cell
FADH2: Flavin Adenine Dinucleotide
fDEGs: Fasting differential genes
FIS1: Fission 1 Protein
GYP: Gypenosides
GTP: Guanosine triphosphate
HCC: hepatocellular carcinoma
HK: Hexokinase
HR1: Hemagglutinin Receptor 1
HSCTe: Human Stem Cell Transplantation
HSP27: Heat Shock Protein 27
IA: Ilexgenin A
IMM: Inner Mitochondrial Membrane
KRG: Korean Red Ginseng
MTFP1: mitochondrial fission process 1
mTORC1: mammalian targeting Rapamycin complex 1
mPTP: mitochondrial permeability transition pore
MAMs: Mitochondria Associated Membranes
MCU: mitochondrial calcium unidirectional transporter
MCAO: Middle cerebral artery occlusion mice
MUL1: Mitochondrial E3 Ubiquitin Protein Ligase 1
MKK: Mitogen-Activated Protein Kinase Kinase
MAPK: Mitogen-Activated Protein Kinase
NADH: Nicotinamide Adenine Dinucleotide, Reduced Form
nDNA: nuclear DNA
NSCLC: Non-small cell lung cancer
NAC: N-Acetylcysteine
OXPHOS: oxidative phosphorylation
NLRP3: NOD-like Receptor Family Pyrin Domain Containing 3
OMM: Outer Mitochondrial Membrane
OSCC: Oral Squamous Cell Carcinoma
PrP1: Prion Protein 1
PTSD: Post-Traumatic Stress Disorder
PI3k: Phosphoinositide 3-Kinase
PDK1: 3-Phosphoinositide-Dependent Protein Kinase 1
ROS: Reactive Oxygen Species
ROS: Reactive oxygen species
RGCs: Retinal ganglion cells
SMEM: Small Molecule Exchange Membrane
SMURF1: Smad Ubiquitin Ligase E3
SRC: Src Proto-Oncogene, Non-Receptor Tyrosine Kinase
TCA: Tricarboxylic Acid Cycle
TM: Transmembrane
TME: tumor microenvironment
TSC1: Tuberous Sclerosis Complex 1
RPKM: Reads Per Kilobase of exon model per Million mapped reads
RhEb: Ras Homolog Enriched in Brain
TP: Testosterone propionate
VRAP: Vacuolar ATPase Assembly Protein
